# Machine learning‐based estimation of patient body weight from radiation dose metrics in computed tomography

**DOI:** 10.1002/acm2.14467

**Published:** 2024-07-23

**Authors:** Hajime Ichikawa, Shota Ichikawa, Yasuhiro Sawane

**Affiliations:** ^1^ Department of Radiology Toyohashi Municipal Hospital Toyohashi Aichi Japan; ^2^ Department of Quantum Medical Technology Institute of Medical Pharmaceutical and Health Sciences Kanazawa University Kanazawa Ishikawa Japan; ^3^ Department of Radiological Technology School of Health Sciences Faculty of Medicine Niigata University Niigata Japan; ^4^ Institute for Research Administration Niigata University Niigata Japan

**Keywords:** body weight estimation, computed tomography, dose metrics, machine learning, radiation dose management

## Abstract

**Purpose:**

Currently, precise patient body weight (BW) at the time of diagnostic imaging cannot always be used for radiation dose management. Various methods have been explored to address this issue, including the application of deep learning to medical imaging and BW estimation using scan parameters. This study develops and evaluates machine learning‐based BW prediction models using 11 features related to radiation dose obtained from computed tomography (CT) scans.

**Methods:**

A dataset was obtained from 3996 patients who underwent positron emission tomography CT scans, and training and test sets were established. Dose metrics and descriptive data were automatically calculated from the CT images or obtained from Digital Imaging and Communications in Medicine metadata. Seven machine‐learning models and three simple regression models were employed to predict BW using features such as effective diameter (ED), water equivalent diameter (WED), and mean milliampere‐seconds. The mean absolute error (MAE) and correlation coefficient between the estimated BW and the actual BW obtained from each BW prediction model were calculated.

**Results:**

Our results found that the highest accuracy was obtained using a light gradient‐boosting machine model, which had an MAE of 1.99 kg and a strong positive correlation between estimated and actual BW (*ρ = *0.972). The model demonstrated significant predictive power, with 73% of patients falling within a ±5% error range. WED emerged as the most relevant dose metric for BW estimation, followed by ED and sex.

**Conclusions:**

The proposed machine‐learning approach is superior to existing methods, with high accuracy and applicability to radiation dose management. The model's reliance on universal dose metrics that are accessible through radiation dose management software enhances its practicality. In conclusion, this study presents a robust approach for BW estimation based on CT imaging that can potentially improve radiation dose management practices in clinical settings.

## 1 INTRODUCTION

Patient body weight (BW) is a pivotal metric in medical practice, particularly in the domains of radiology and nuclear medicine. In these fields, patient BW is used to determine pharmacologic stress and the doses of contrast media and radiopharmaceuticals. Patient BW is critical to the management of radiation doses through the use of diagnostic reference levels by defined weight groups.^1^ When BW measures are unavailable or impractical, BW must be accurately estimated for proper radiation dose management, a challenge that has recently received much attention.

Patient BW at the time of diagnostic imaging is rarely accurately recorded in hospital information systems or Digital Imaging and Communications in Medicine (DICOM) metadata. When BW is registered and can be used for radiation dose management, the recorded measurement must be used even when it is not apparent when the measurement was taken. In some instances, this may have been several years before the radiological imaging. For emergency care, visual BW estimation by healthcare staff can be necessary if the patient is unresponsive and there is no alternative means of measurement, but its reliability is limited.^2^, ^3^ To overcome these limitations, BW estimation methods using medical images or their acquisition parameters have been investigated.^4^, ^5^, ^6^, ^7^, ^8^, ^9^, ^10^ Chest x‐ray images,^8^ computed tomography (CT) scout images of the chest and abdomen,^7^, ^9^, ^10^ and CT cross‐sectional images^4^, ^5^, ^6^ have been mainly used to estimate patient BW. Specifically, patient BW has been estimated using multiple linear regression analysis based on the size of the vertebral body and abdomen measured from the CT image at the first lumbar level,^4^ by multiplying volumes from whole‐body CT data by a factor,^5^ or by deep learning‐based methods using two‐dimensional images.^7^, ^8^, ^9^, ^10^ Based on CT dose modulation, the effective tube‐current time product, in milliampere‐seconds (mAs), has been used as a dose indicator for BW estimation according to the dose report automatically created by the CT scanner.^6^ Moreover, research has shown that effective diameter (ED),^11^, ^12^ water equivalent diameter (WED),^13^ volume CT dose index (CTDI_vol_),^14^ and size‐specific dose estimates (SSDE)^15^ are all strongly correlated with BW.

CT scout images provide accessible data, as they are almost always acquired to plan and delimit subsequent CT examinations. However, they can contain errors due to patient miscentering.^16^ Previous research has shown that deep‐learning prediction models based on two‐dimensional images have relatively high accuracy,^8^ but difficulties with model versatility make their universal clinical use problematic. BW estimates based on the manual measurement of a patient's organ size^4^, ^17^ have high operator burden and poor reproducibility. Dose metrics such as ED, WED, SSDE, and CTDI_vol_ can be used in automated calculations using radiation dose management software, which has become popular in recent years;^11^, ^12^, ^13^, ^16^ however, the accuracy of BW prediction models based on simple regressions from a single dose metric is limited. Therefore, this study hypothesizes that patient BW can be estimated with high accuracy using a regression model that utilizes multiple radiation dose‐related variables obtained from CT images by radiation dose management software. To test this hypothesis, we developed seven machine learning‐based patient BW prediction models using 11 radiation dose‐related variables. For comparative purposes, we set three simple regressions, each from a single dose metric as a reference standard. The purpose of the study was to evaluate the accuracy of these models and assess their potential for clinical use.

## 2 METHODS

### 2.1 Patients and PET/CT scans

The study participants comprised 3996 consecutive patients who underwent ^18^F‐fluorodeoxyglucose (^18^F‐FDG) positron emission tomography with computed tomography (PET/CT) scans from the skull base to the mid‐thigh (441.2 ± 41.8 slices) between April 2020 and October 2023 in hospital A (blinded for review). The patient demographics are shown in Table 1. All patients fasted for at least 6 h before their PET/CT scan, were measured for height and BW, and administered ^18^F‐FDG (approximately 3.7 MBq/kg) approximately 1 h before the scan. They then rested for 50 min before undergoing their PET/CT scan, which was performed using a Biograph mCT Flow 20 VG62C (Siemens Healthineers, Erlangen, Germany) scanner. Weighing scales with a height meter (MS4900 + HM200D, AS ONE, Osaka, Japan) were used for the height and weight measurements. These are calibrated annually at our facility. The scans were performed at a kVp of 120, with an effective mAs of 45, 2 mm slices, 0.5 s/rotation, and 1.2 pitch. The reconstruction kernels were created using an I36f heartview medium ASA. Our training data were obtained from the medical records of patients who underwent these scans at our facility between April 2020 and August 2023 (*n* = 3785) and our test data from those scanned between September and October 2023 (*n* = 211). The training set was used to train the models in patient BW estimation. The test set was used to measure the performance of the models. The study was conducted in accordance with the tenets of the 2013 revision of the Declaration of Helsinki and approved by the ethics committee of Toyohashi municipal hospital. Informed consent was obtained from the patients in the form of an opt‐out (approval no: 772).

**TABLE 1:** Patient demographics.

	Training		Test	
	Mean	SD	Mean	SD
*N*	3785		211	
Age (y)	69.5	11.7	70.0	12.6
Height (cm)	160.6	9.1	160.0	9.0
Weight (kg)	57.8	12.3	56.3	10.9
BMI (kg/m^2^)	22.3	3.9	21.9	3.4
Sex (female/male)	1521 / 2269		83 / 128	

Abbreviations: BMI, body mass index; SD, standard deviation.

No significant differences in these variables were found between the training and test datasets.

### 2.2 Dose metrics and descriptive data

The dose metrics and descriptive data were either obtained from DICOM metadata or automatically calculated from the CT images using radiation dose monitoring software (Radimetrics, Bayer HealthCare, Whippany, NJ, USA). The 10 dose metrics and descriptive data obtained for the BW prediction were: effective diameter (ED, mm),^18^ water equivalent diameter (WED, mm),^19^ mean milliampere‐seconds (mAs_mean_), maximum mAs (mAs_max_), scan length (mm), size‐specific dose estimate (SSDE, mGy),^18^ age at examination (*y*), sex, volume computed tomography dose index (CTDI_vol_, mGy),^20^ and dose‐length product (DLP) (mGy cm). For continuous data, data standardization was performed to achieve a mean of zero and a standard deviation of 1 before inputting the features into the models. Categorical data (i.e., sex) were coded using the one‐hot method (i.e., for each category of female (F) and male (M), 1 was assigned if they matched and 0 if they did not). A total of 11 features were used to train the models to predict patient BW.

### 2.3 Body weight prediction models

Based on the previous literature,^6^, ^11^, ^13^ we constructed three simple linear regression models using one input variable for each. The variables used were ED, WED, and mAs_mean_. These models were used as reference standards. We then created seven different supervised machine‐learning models: linear, ridge, least absolute shrinkage selector operator (LASSO), elastic net, decision trees, random forest, and light gradient‐boosting machine (LightGBM) to predict patient BW using 11 input variables derived from the radiation dose monitoring software applied to patient imaging data. These machine‐learning models are considered well‐suited to classification and regression tasks in medicine.^21^, ^22^

Multiple linear regression and its penalized versions (ridge, LASSO, and elastic net) were selected as the linear regression models. The linear regression model is typically expressed using Equation (1):

(1) $$y_{i}=\beta_{0}+\sum_{j=1}^{n}\beta_{j}x_{ij}$$

where *y_i_* is the continuous outcome value of subject i, β_0_ is the intercept, β_j_ is the coefficient of feature j, and *x*_ij_ is feature j of subject i.

The regression parameter of a linear regression model is determined using the least squares method by minimizing the error term in the unknown β_j_ (Equation (2)).

(2) $$\beta =argmin_{\beta }\left\{\frac{1}{n}\sum_{i=1}^{n}{\left(y_{i}-{\hat{y}}_{i}\right)}^{2}\right\}$$

Ridge regression^23^ is used to reduce model complexity through L2 regularization. LASSO regression^23^ incorporates L1 regularization. It also uses shrinkage as a regularization technique to reduce overfit and simplifies the model by reducing the coefficients of less influential variables to zero or toward it. Elastic net regression^24^ combines the LASSO and ridge regression methods to produce results that handle bias better than LASSO or ridge regression. The regression parameters for ridge, LASSO, and elastic net are estimated using Equations ((3), (4), (5)), respectively:

(3) $$\beta =argmin_{\beta }\left\{\frac{1}{n}\sum_{i=1}^{n}{\left(y_{i}-{\hat{y}}_{i}\right)}^{2}+\lambda \sum_{j=1}^{p}\beta_{j}^{2}\right\}$$

(4) $$\beta =argmin_{\beta }\left\{\frac{1}{n}\sum_{i=1}^{n}{\left(y_{i}-{\hat{y}}_{i}\right)}^{2}+\lambda \sum_{j=1}^{p}\left|\beta_{j}\right|\right\}$$

(5) $$\beta =argmin_{\beta }\left\{\frac{1}{n}\sum_{i=1}^{n}{\left(y_{i}-{\hat{y}}_{i}\right)}^{2}+\lambda_{1}\sum_{j=1}^{p}\beta_{j}^{2}+\lambda_{2}\sum_{j=1}^{p}\left|\beta_{j}\right|\right\}$$

where *λ* is the regularization parameter to be optimized.

Three common nonlinear regression models were selected: decision trees, random forest, and LightGBM. Decision trees for regression^25^ so named for their tree‐like structure, are capable of predicting the value of a target variable (in this case, patient BW) by learning straightforward decision rules derived from data features. Random forest^26^ employs an ensemble learning technique, constructing multiple decision tree regressors on various sub‐samples of the dataset and averaging to enhance predictive accuracy while controlling overfit. LightGBM,^27^ a library developed by Microsoft, efficiently implements a gradient‐boosting model. It inherits high predictivity while addressing scalability and issues with long computation time through the adoption of a leaf‐wise tree growth strategy and the introduction of novel techniques.

The analyses were performed using *Scikit‐learn* packages (https://scikit‐learn.org/stable/index.html) in Python on a standard personal computer without a graphics processing unit. The hyperparameters were determined for the training data using a grid search with fivefold cross‐validation to determine which set of hyperparameters produced the lowest mean absolute error. The hyperparameter tuning used with each model is summarized in Table S1.

### 2.4 Comparisons between actual and estimated body weights

The performance of the models was evaluated using test sets. The mean absolute error (MAE), root mean squared error (RMSE), Spearman's rank correlation coefficient, and percent difference (%Dif) were used to compare the BWs obtained from each estimation model with the actual BWs. The MAE, RMSE, and %Dif were calculated using Equations ((6), (7), (8)), respectively.

(6) $$\begin{array}{c} \mathrm{MAE}=\frac{1}{N}\sum_{i=1}^{N}\left|y_{i}-\overset{´}{y}i\right| \end{array}$$

(7) $$\begin{array}{c} \mathrm{RMSE}=\sqrt{\frac{1}{N}\sum_{i=1}^{N}{\left(y_{i}-\overset{´}{y}i\right)}^{2}} \end{array}$$

(8) $$\%\mathrm{Dif}=\frac{y_{i}-\overset{´}{y}i}{y_{i}}\;\times \;100$$

where *N* is the total number of subjects, $y_{i}$ is the actual BW, and $\overset{´}{y}i$ is the estimated BW. The actual and estimated BWs were compared by linear regression analysis and systematic differences were detected by Bland–Altman analysis.

### 2.5 Model interpretation with Shapley additive explanations

The Shapley additive explanations (SHAP) approach,^28^ an extension of the Shapley values from game theory, was employed to elucidate the importance value assigned to each feature for individual predictions in the test data. These values are computed based on the difference in predictions when all features are used compared to using only a subset.

### 2.6 Statistical analyses

All data were evaluated for the normality of their distribution with the Shapiro‐Wilk test. The Mann–Whitney *U*‐test was used to analyze unpaired data. Nominal variables were tested using the chi‐square test. Actual and estimated BW were compared using the Friedman test, and the Bonferroni method was used as a post hoc test. Spearman's rank correlation coefficient was used to determine the relationships between actual and estimated BWs. All statistical analyses were performed using SPSS v. 27 (IBM Corp., Armonk, NY, USA) software. *p*‐Values of <0.05 were considered statistically significant.

## 3 RESULTS

The dose metrics obtained by the radiation dose monitoring software are shown in Table 2. There was a significant difference in the SSDE between the training and test data (*p* = 0.02), but not in the other dose metrics. Scatter plots showing the relationships of actual BW with ED, WED, and mAs_mean_ used to develop the simple regression models are shown in Figure S1. Seven models were developed for BW estimation using the dose metrics from the training data.

**TABLE 2:** Radiation dose metrics in a training set and a test set calculated using dose monitoring software.

	Training		Test	
	Mean	SD	Mean	SD
ED	251.4	25.5	252.3	22.7
WED	241.9	23.1	239.7	21.4
Average mAs	52.9	13.5	52.3	16.3
Maximum mAs	93.0	37.4	107.8	44.5
Scan length	893.3	83.6	887.9	66.4
SSDE^*^	5.0	1.0	5.0	1.4
CTDIvol	3.5	0.9	3.5	1.1
DLP	317.4	95.2	311.7	108.3

* Significant difference (*p* = 0.02).

**FIGURE 1:**
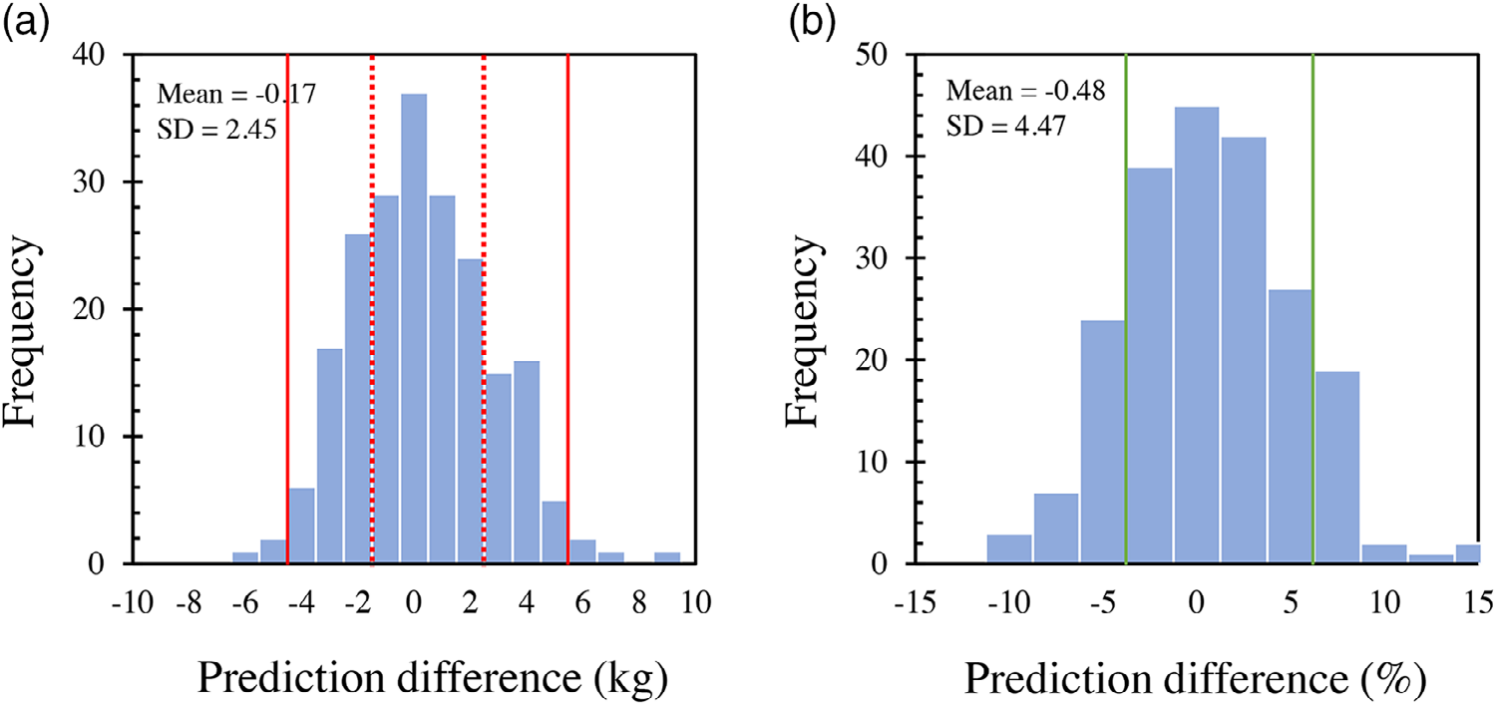
Histograms showing the differences between actual patient body weights and the body weight estimations of our LightGBM machine‐learning model. The x‐axes represent (a) weight and (b) percentage. The solid and dashed red lines in (a) represent the ±5 kg and ±2 kg ranges, respectively. The solid green lines in (b) represent the ±5% range.

**FIGURE 2:**
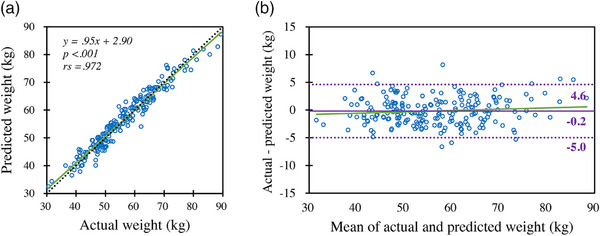
Scatter plot and Bland–Altman plot showing the relationships between actual patient body weights and the body weight estimations of a LightGBM machine‐learning model. (a) Correlations between actual and estimated body weights. The green solid line is the regression line; the black dotted line is the identity line. (b) Bland–Altman plot comparing the actual and estimated body weights. The solid purple line shows mean differences; the two dashed purple lines show the 95% confidence intervals.

Table 3 summarizes the accuracy levels of the BW estimates from the three simple regression and the seven machine‐learning models. The MAE and the RMSE were lowest in the LightGBM model, and the mAs_mean_ had the highest value. The Spearman's rank correlation coefficients for actual and estimated BW for all predictive models demonstrated a strong positive correlation, with LightGBM having the strongest correlation. The MAE of the BW estimate by the LightGBM model was 1.99 kg, with 56% and 97% of patients within prediction difference ranges of ±2 and ±5 kg, respectively (Figure 1a). There was a percentage difference of within ±5% for 73% of the patients (Figure 1b). All six patients with an absolute difference >10% had low BWs (41−62 kg). The correlation coefficient between actual BW and the estimated BW obtained by the LightGBM model was *ρ = *0.972, indicating a strong positive correlation (Figure 2a). Our Bland–Altman analysis comparing actual and predicted BW (Figure 2b) showed neither fixed nor proportional errors in the LightGBM model (95% confidence interval −5.0, 4.6).

**TABLE 3:** Error and Spearman's rank correlation coefficients between the mean actual and estimated body weights for estimations made by different machine‐learning models.

Model		MAE (kg)		RMSE (kg)	*ρ*
Simple regression	Effective diameter	3.69	(0.0−15.2)	4.79	0.913
	WED	3.22	(0.0−14.7)	4.18	0.923
	Mean mAs	5.28	(0.0−49.3)	7.55	0.851
Linear regression	Linear regression	2.01	(0.0−9.4)	2.56	0.973
	Ridge	2.03	(0.0−9.1)	2.58	0.973
	LASSO	2.03	(0.0−9.1)	2.58	0.973
	Elastic net	2.03	(0.0−9.1)	2.58	0.973
Nonlinear regression	Decision tree	2.65	(0.1−8.7)	3.24	0.958
	Random forest	2.11	(0.0−8.4)	2.66	0.970
	LightGBM	1.99	(0.0−8.2)	2.45	0.972

Weights are given as means and ranges. *ρ*, Spearman's rank correlation coefficient.

No fixed errors were identified in any of the models by the Bland–Altman analyses, but proportional errors were observed in the simple linear regression prediction models that used ED and mAs_mean_ (Figure S2). Compared to LightGBM, the random forest model was almost identically distributed, but the histogram of the decision tree model exhibited a wider distribution (Figure S3). The results from the four linear regression models were almost identical in the histograms, with slightly wider distributions than the LightGBM model. The histograms of the simple regression models showed even wider distributions.

**FIGURE 3:**
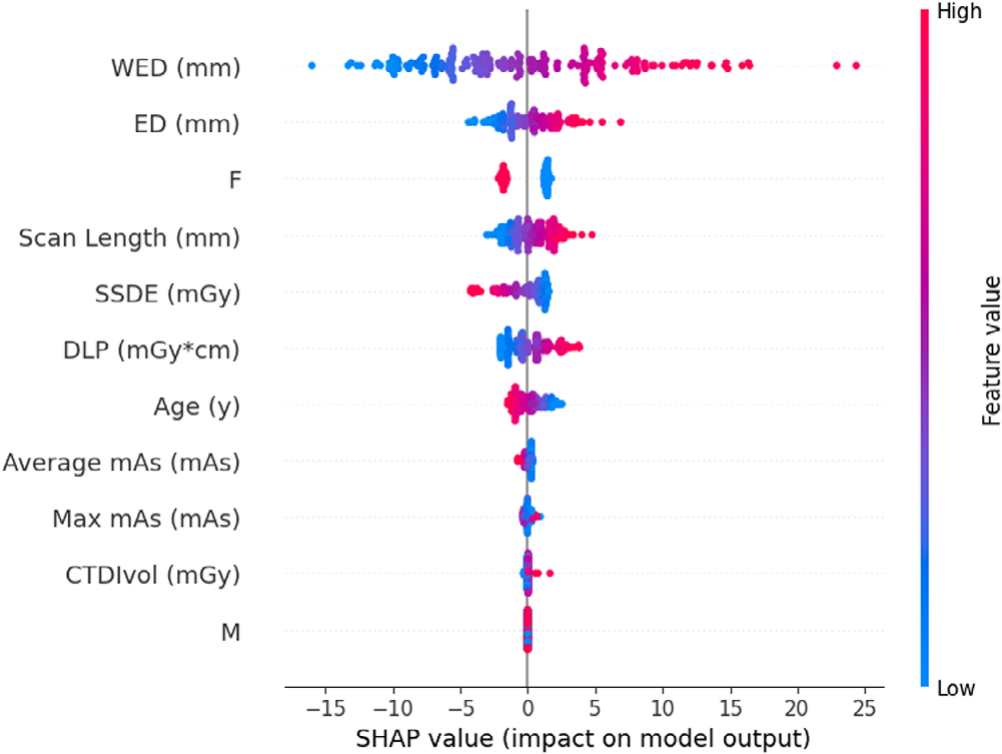
The SHAP value features used for body weight estimation in a LightGBM machine‐learning model. The plot shows the 11 features evaluated using the SHAP method and the effects of each feature on the body weight estimations of the model. F, female; M, male.

To visually explain the relevance of the different dose metrics to BW estimation, Figure 3 shows the SHAP values obtained for the LightGBM model, which had the highest accuracy. The dose metrics are shown from top to bottom in order of importance for BW estimation. Feature values are color‐coded, with red data points indicating higher values and blue data points indicating lower values. The horizontal axis indicates the relationship of the SHAP values to the patient BW estimation for each subject. WED was found to make the greatest contribution to BW estimation, followed by ED and patient sex. The higher values for WED and ED represent their stronger influence on higher BW estimates while being female had a stronger influence on lower BW estimates.

## 4 DISCUSSION

In this study, we tested the accuracy of 10 prediction models using 11 radiation dose features to accurately estimate a patient's BW from dose metrics when it is unknown but required for radiation dose management. We found that a LightGBM prediction model provided the most accurate estimates of BW and that WED was the dose metric that contributed most to estimation accuracy. This prediction model can estimate BW with an extremely high accuracy sufficient for its application in radiation dose management. The MAE and correlation coefficients of the LightGBM prediction model were 1.99 kg and 0.972, respectively, and the estimates for 73% of the patients were within an error range of ±5%. To the best of our knowledge, this is the most accurate prediction model currently available.

Previous studies have reported BW to be strongly correlated with ED (*r *= 0.805−0.929), WED (*r* ^2^ = 0.43−0.55), and effective mAs (*r* = 0.969),^6^, ^11^, ^13^ and BW prediction models using these variables as the single metric can provide simple and immediate BW estimations as ED and WED can easily be obtained from scout images or cross‐sectional data.^14^, ^18^ However, we found errors >5% in 55% of the estimates obtained using the ED simple regression model and 47% of the estimates by the WED simple regression model. Thus, the accuracy of BW predictions obtained from these single‐metric models is limited. An average difference of 5 cm in the EDs obtained from chest and abdominal CT scans has previously been reported.^12^ The scan range also seems to affect the accuracy of BW estimation and the consistency of the scan range of our CT data may partially explain the high accuracy of the BW estimations with the seven regression models.

Previous studies have explored deep‐learning prediction models that use chest radiographs and CT scout images of the chest or abdomen.^7^, ^8^, ^9^, ^10^ Of these, the deep‐learning BW prediction model based on chest x‐ray images^8^ is superior as this type of imaging is available at all medical institutions, and the MAE for women and men are 2.63 and 3.35 kg, respectively. However, these estimation models utilize data from patients’ medical checkups, which may deviate from clinical data. The estimation models developed in the present study are based on clinical data. For example, a patient with pleural effusion may have the same ED as healthy patients but a different WED. This was accounted for in our study data. BW prediction models that utilize data from CT scout images of the chest and abdomen or chest x‐rays are considered particularly useful with pediatric patients,^10^ in whom there are marked changes in BW as they grow but their accuracy is relatively poor, with MAE of 4 kg. Although deep‐learning models seem to have less error than single‐metric regression models, obtaining the necessary data from DICOM images is a challenging task. Moreover, ED values obtained from scout images may vary in accuracy due to patient miscentering.^16^ Similarly, the accuracy of WED calculations has been reported to be higher when the data come from diagnostic rather than scout CT images.^29^ We believe our prediction model solves the aforementioned problems with other models.

A further reason for the superiority of the model presented in this study is the use of 11 dose metrics derived from over 400 CT slices. Although this may seem to complicate the development of the prediction model, these universal metrics can be easily obtained using radiation dose management software already commonly used in medical institutions. Hence, the model should be relatively easy to apply as the relevant medical personnel will likely be familiar with the software. The construction of our proposed model is feasible on a standard personal computer and does not require advanced computational resources, such as a graphics processing unit. We believe that the development of our prediction model through machine learning using only numerical values adds further value since it can be rapidly produced on a general‐purpose computer. It is laborious and time‐consuming to perform BW measurements before every CT examination and correctly register the results as DICOM metadata and the delay decreases the throughput of the examinations. Yet, when a patient's BW is obtained from modality worklist management, there is often no record of when the data were obtained, and these data are infrequently updated, regardless of variations in the patient's BW. Furthermore, patients themselves are not always aware of their current correct BW. We believe that the BW prediction model presented in this study is sufficiently accurate to have great utility in radiation dose management. In the future, if our proposed prediction model is integrated into radiation dose management software, the clinical workflow in radiation dose management will be advanced.

Among the 11 dose metrics used, WED, ED, sex, scan length, and SSDE were the five most relevant (Figure 3). The correlation coefficient of a simple regression between ED and BW has been reported to be approximately 0.725−0.929,^11^, ^12^ which is roughly equivalent to the result we obtained from our simple ED regression model (ρ = 0.913). Previous studies on BW estimation using dose‐modulated mAs with postmortem CT imaging have suggested that higher correlations can be obtained with CT images from living patients.^6^ However, we found overestimations exceeding 45% in a few patients. This difference may have been caused by the high mAs parameters given in postmortem CT protocol^29^ versus the low mAs parameters advised for clinical PET. The use of dose metrics obtained from diagnostic CT images may have affected the contribution of each metric to the BW estimations, resulting in higher accuracy. However, the MAE for patient BW estimation from a single CT image at the level of the first lumbar vertebra is approximately 5 kg for both men and women,^4^ which is significantly higher than the MAE of our predicted BW based on our low mAs CT. Therefore, the efficacy of our BW prediction model is likely higher than would be possible due only to differences in the reference mAs of the CT scan parameters.

This study had several limitations. Our prediction model used 11 radiation dose metrics, necessitating a radiation dose management system. The BW prediction model in this study was developed using low‐dose CT images based on the PET/CT scan protocol and its accuracy when using diagnostic CT images was not investigated. However, since the tube voltages we set were in the range of standard diagnostic CT protocols, we believe that the use of diagnostic CT images for BW estimation would not reduce the accuracy of the model.^30^ Nevertheless, further validation is required. Also, our proposed prediction model was developed from patients who underwent PET/CT on a single machine at our institution, which potentially limits the generalizability of the model. Future validation with independent datasets from different facilities and CT systems would allow for clinical use. Because WED is the major radiation dose metric in our proposed prediction model, this variable may be overestimated in patients who only undergo contrast‐enhanced CT, reducing the accuracy of the BW estimation.^31^ However, patients who undergo contrast‐enhanced CT were not the target population of this study because, in these patients, BW is measured to determine the dose of contrast media. We also did not investigate the use of CT data from the chest or abdomen only.

## 5 CONCLUSION

In this study, we developed seven BW estimation models and found our LightGBM model, which uses multiple radiation dose variables, to be the most accurate. The validity of the proposed model is supported by the fact that WED and ED, which are known to be strongly correlated with BW, were found to have a strong influence on BW estimation. Our results demonstrate that the proposed model is sufficiently accurate for the estimation of patient BW for radiation dose management.

## AUTHOR CONTRIBUTIONS

**Hajime Ichikawa**: Conceptualization; data curation; writing—original draft; formal analysis; project administration. **Shota Ichikawa**: Methodology; software; validation; resources; writing—original draft; funding acquisition. **Yasuhiro Sawane**: Investigation; data curation; writing—original draft.

## CONFLICT OF INTEREST STATEMENT

The authors declare no conflicts of interest.

## Supporting information

Supporting information

Supporting information

## Data Availability

The code generated during the current study is available from the corresponding author upon reasonable request. However, the authors do not currently have permission to make the data publicly available.

## References

[1] Vassileva J , Rehani M . Diagnostic reference levels. AJR Am J Roentgenol. 2015;204(1):W1‐W3. 25539261 10.2214/AJR.14.12794

[2] Anglemyer BL , Hernandez C , Brice JH , Zou B . The accuracy of visual estimation of body weight in the ED. Am J Emerg Med. 2004;22(7):526‐529. 15666254 10.1016/j.ajem.2004.09.002

[3] Hall WL 2nd , Larkin GL , Trujillo MJ , Hinds JL , Delaney KA . Errors in weight estimation in the emergency department: comparing performance by providers and patients. J Emerg Med. 2004;27(3):219‐224. 15388205 10.1016/j.jemermed.2004.04.008

[4] Geraghty EM , Boone JM . Determination of height, weight, body mass index, and body surface area with a single abdominal CT image. Radiology. 2003;228(3):857‐863. 12881576 10.1148/radiol.2283020095

[5] Jackowski C , Schwendener N , Zeyer‐Brunner J , Schyma C . Body weight estimation based on postmortem CT data–validation of a multiplication factor. Int J Legal Med. 2015;129(5):1121‐1125. 26003443 10.1007/s00414-015-1199-x

[6] Gascho D , Ganzoni L , Kolly P , et al. A new method for estimating patient body weight using CT dose modulation data. Eur Radiol Exp. 2017;1(1):23. 29708203 10.1186/s41747-017-0028-z PMC5909357

[7] Ichikawa S , Hamada M , Sugimori H . A deep‐learning method using computed tomography scout images for estimating patient body weight. Sci Rep. 2021;11(1):15627. 34341462 10.1038/s41598-021-95170-9 PMC8329066

[8] Ichikawa S , Itadani H , Sugimori H . Prediction of body weight from chest radiographs using deep learning with a convolutional neural network. Radiol Phys Technol. 2023;16(1):127‐134. 36637719 10.1007/s12194-023-00697-3

[9] Ichikawa S , Itadani H , Sugimori H . Deep learning‐based body weight from scout images can be an alternative to actual body weight in CT radiation dose management. J Appl Clin Med Phys. 2023;24(8):e14080. 37337623 10.1002/acm2.14080 PMC10402676

[10] Demircioglu A , Quinsten AS , Umutlu L , Forsting M , Nassenstein K , Bos D . Determining body height and weight from thoracic and abdominal CT localizers in pediatric and young adult patients using deep learning. Sci Rep. 2023;13(1):19010. 37923758 10.1038/s41598-023-46080-5 PMC10624655

[11] Fukunaga M , Matsubara K , Ichikawa S , Mitsui H , Yamamoto H , Miyati T . CT dose management of adult patients with unknown body weight using an effective diameter. Eur J Radiol. 2021;135:109483. 33388531 10.1016/j.ejrad.2020.109483

[12] Tessa SC , Seetharam CC , William WB . How inaccurate is weight as a metric for patient size? Comparing patient weight to effective diameter for size‐specific dose estimation. Proc SPIE 2013;8674:867408.

[13] Wati AL , Anam C , Nitasari A , Syarifudin S , Dougherty G . Correlations between body weight and size‐specific dose estimate on thoracic computed tomography examination. Atom Indonesia. 2022;48(1):61‐65.

[14] Christner JA , Braun NN , Jacobsen MC , Carter RE , Kofler JM , McCollough CH . Size‐specific dose estimates for adult patients at CT of the torso. Radiology. 2012;265(3):841‐847. 23091173 10.1148/radiol.12112365

[15] Khawaja RD , Singh S , Vettiyil B , et al. Simplifying size‐specific radiation dose estimates in pediatric CT. AJR Am J Roentgenol. 2015;204(1):167‐176. 25539253 10.2214/AJR.13.12191

[16] Cournane S , Brunell E , Rowan M . Establishing how patient size and degree of miscentring affect CTDI(vol), using patient data from a dose tracking system. Br J Radiol. 2019;92(1099):20180992. 31112413 10.1259/bjr.20180992 PMC6636259

[17] Zopfs D , Theurich S , Große Hokamp N , et al. Single‐slice CT measurements allow for accurate assessment of sarcopenia and body composition. Eur Radiol. 2020;30(3):1701‐1708. 31776743 10.1007/s00330-019-06526-9

[18] McCollough C , Leng S , Yu L , et al. Size-specific dose estimates (SSDE) in pediatric and adult body CT examination. AAPM Report. American Association of Physicists in Medicine ; 2016.

[19] McCollough CH , Donovan MB , Bostani M , Brady S , Boedeker K , Boone JM . Use of water equivalent diameter for calculating patient size and size‐specific dose estimates (SSDE) in CT. College Park, MD: American Association of Physicists in Medicine ; 2014. PMC4991550 27546949

[20] McCollough CH , Cody D , Edyvean S , et al. Aapm Report 96: The Measurement, Reporting, and Management of Radiation Dose in CT. Madison, WI: Medical Physics; 2008.

[21] Mostafaei S , Kazemnejad A , Azimzadeh Jamalkans , et al. Identification of novel genes in human airway epithelial cells associated with chronic obstructive pulmonary disease (COPD) using machine‐based learning algorithms. Sci Rep. 2018;8(1):15775. 30361509 10.1038/s41598-018-33986-8 PMC6202402

[22] Kwon OB , Han S , Lee HY , et al. Prediction of postoperative lung function in lung cancer patients using machine learning models. Tuberc Respir Dis (Seoul). 2023;86(3):203‐215. 37038881 10.4046/trd.2022.0048 PMC10323210

[23] Tibshirani R . Regression Shrinkage and Selection via the Lasso. J Royal Stat Soc Series B (Methodol). 1996;58(1):267‐288.

[24] Zou H , Hastie T . Regularization and variable selection via the elastic net. J R Stat Soc Series B: Stat Methodol. 2005;67(2):301‐320.

[25] Loh W‐Y . Classification and regression trees. WIREs Data Mining Knowledge Discov. 2011;1(1):14‐23.

[26] Breiman L . Random forests. Mach Learn. 2001;45(1):5‐32.

[27] Ke G , Meng Q , Finley T , et al. LightGBM: a highly efficient gradient boosting decision tree. Adv Neural Inform Process Syst. 2017;30:1‐9.

[28] Lundberg SM , Lee S-I . A Unified Approach to Interpreting Model Predictions . In: Neural Information Processing Systems. 2017.

[29] Flach PM , Gascho D , Schweitzer W , et al. Imaging in forensic radiology: an illustrated guide for postmortem computed tomography technique and protocols. Forensic Sci Med Pathol. 2014;10(4):583‐606. 24723662 10.1007/s12024-014-9555-6

[30] McCollough CH , McCollough SL , Schneider JJ , et al. Dependence of water‐equivalent diameter and size‐specific dose estimates on CT tube potential. Radiology. 2022;303(2):404‐411. 35040673 10.1148/radiol.210860 PMC9038617

[31] Viggiano B , Rose S , Szczykutowicz TP . Effect of contrast agent administration on water equivalent diameter in CT. Med Phys. 2021;48(3):1117‐1124. 33440020 10.1002/mp.14721

