# Supplementary material for: Machine learning‐based estimation of patient body weight from radiation dose metrics in computed tomography
Source: J Appl Clin Med Phys. 2024 Jul 23;25(9):e14467. doi: 10.1002/acm2.14467 (PMC11492421; doi:10.1002/acm2.14467)
Supplement: Supplementary file 1 — Supporting information [file ACM2-25-e14467-s002.pptx]

## Slide 1
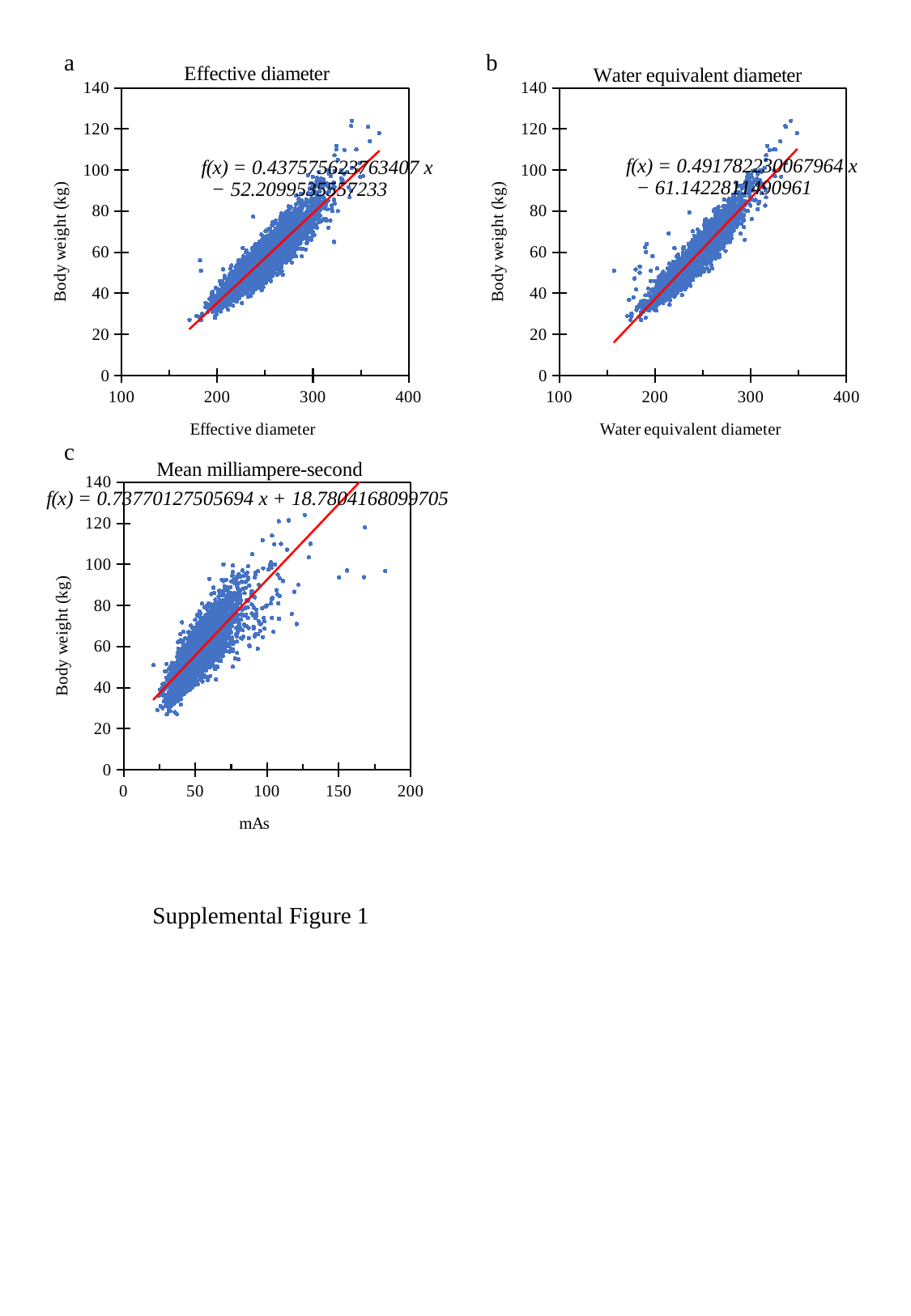

b
a
### Chart: Water equivalent diameter
| Category | 患者の体重 kg |
|---|---|
### Chart: Effective diameter
| Category | 患者の体重 kg |
|---|---|c
### Chart: Mean milliampere-second
| Category | 患者の体重 kg |
|---|---|Supplemental Figure 1

## Slide 2
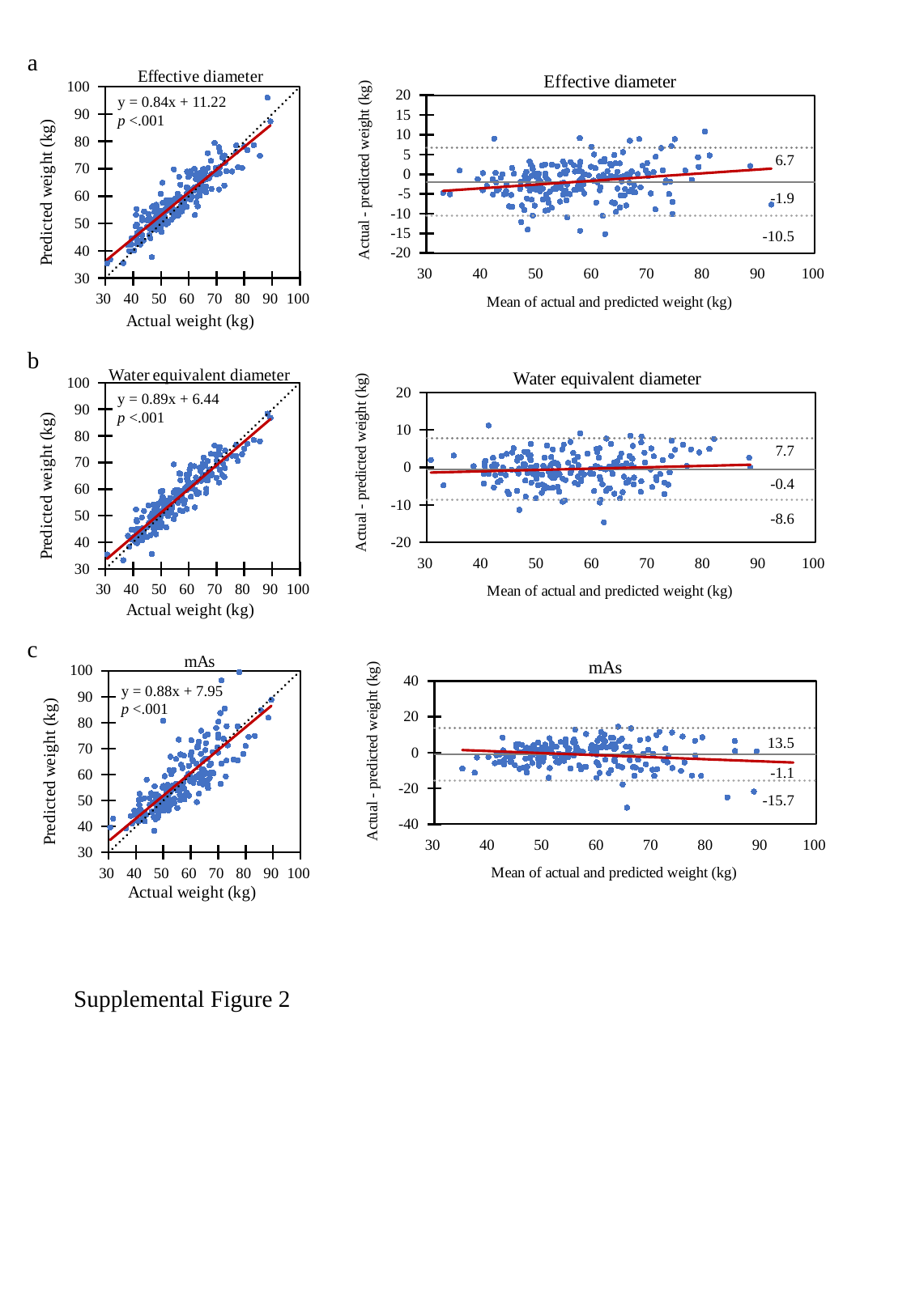

### Chart: Effective diameter
| Category | Mean | |
|---|---|---|
### Chart: Effective diameter
| Category | Mean | | | |
|---|---|---|---|---|a
y = 0.84x + 11.22
p <.001
6.7
-1.9
-10.5
### Chart: Water equivalent diameter
| Category | Mean | |
|---|---|---|
### Chart: Water equivalent diameter
| Category | Mean | | | |
|---|---|---|---|---|b
y = 0.89x + 6.44
p <.001
7.7
-0.4
-8.6
### Chart: mAs
| Category | Mean | | | |
|---|---|---|---|---|
### Chart: mAs
| Category | Mean | |
|---|---|---|c
y = 0.88x + 7.95
p <.001
13.5
-1.1
-15.7
Supplemental Figure 2

## Slide 3
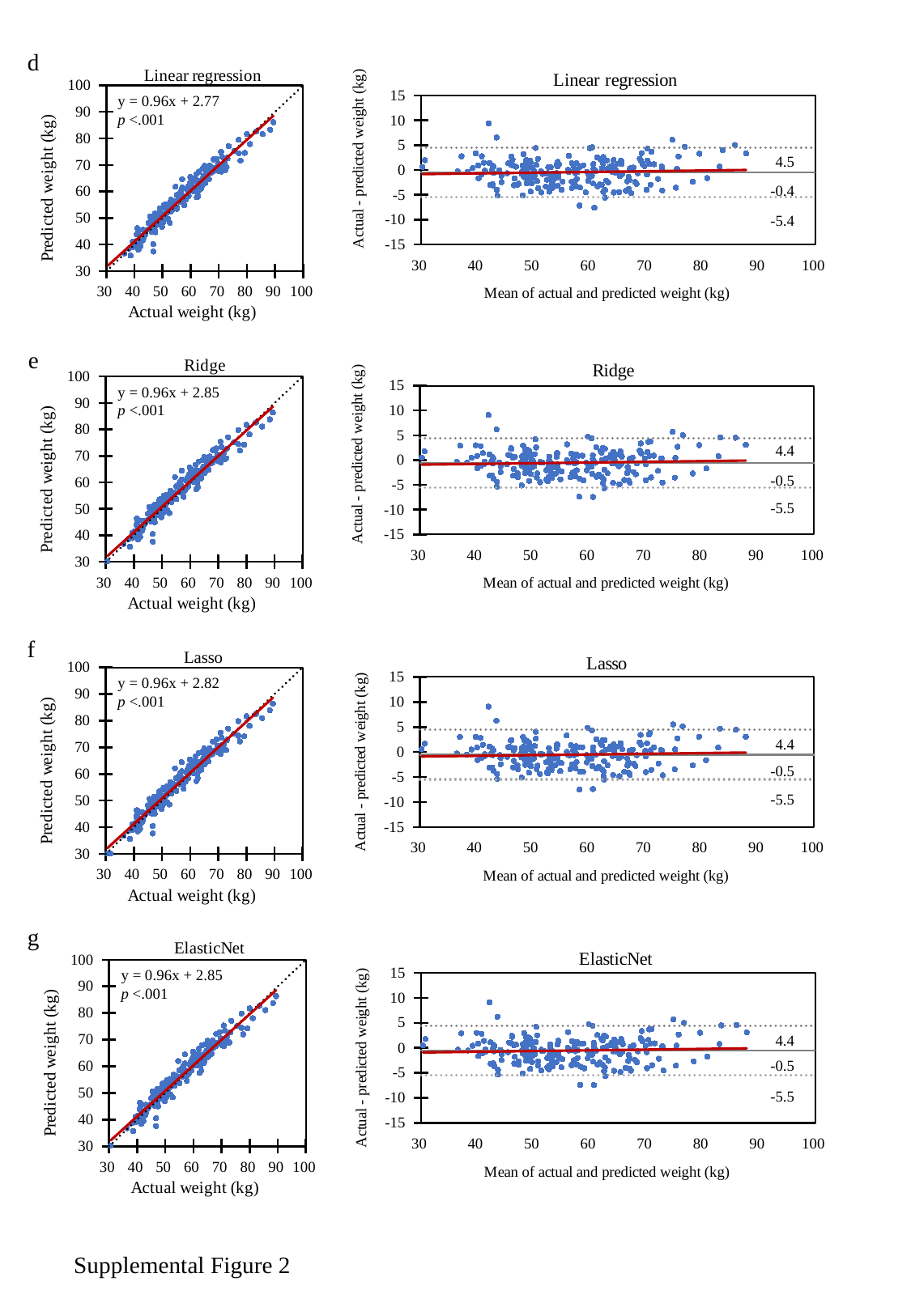

### Chart: Linear regression
| Category | Mean | |
|---|---|---|
### Chart: Linear regression
| Category | Mean | | | |
|---|---|---|---|---|d
y = 0.96x + 2.77
p <.001
4.5
-0.4
-5.4
### Chart: Ridge
| Category | Mean | | | |
|---|---|---|---|---|
### Chart: Ridge
| Category | Mean | |
|---|---|---|e
y = 0.96x + 2.85
p <.001
4.4
-0.5
-5.5
### Chart: Lasso
| Category | Mean | |
|---|---|---|
### Chart: Lasso
| Category | Mean | | | |
|---|---|---|---|---|f
y = 0.96x + 2.82
p <.001
4.4
-0.5
-5.5
### Chart: ElasticNet
| Category | Mean | |
|---|---|---|g
### Chart: ElasticNet
| Category | Mean | | | |
|---|---|---|---|---|y = 0.96x + 2.85
p <.001
4.4
-0.5
-5.5
Supplemental Figure 2

## Slide 4
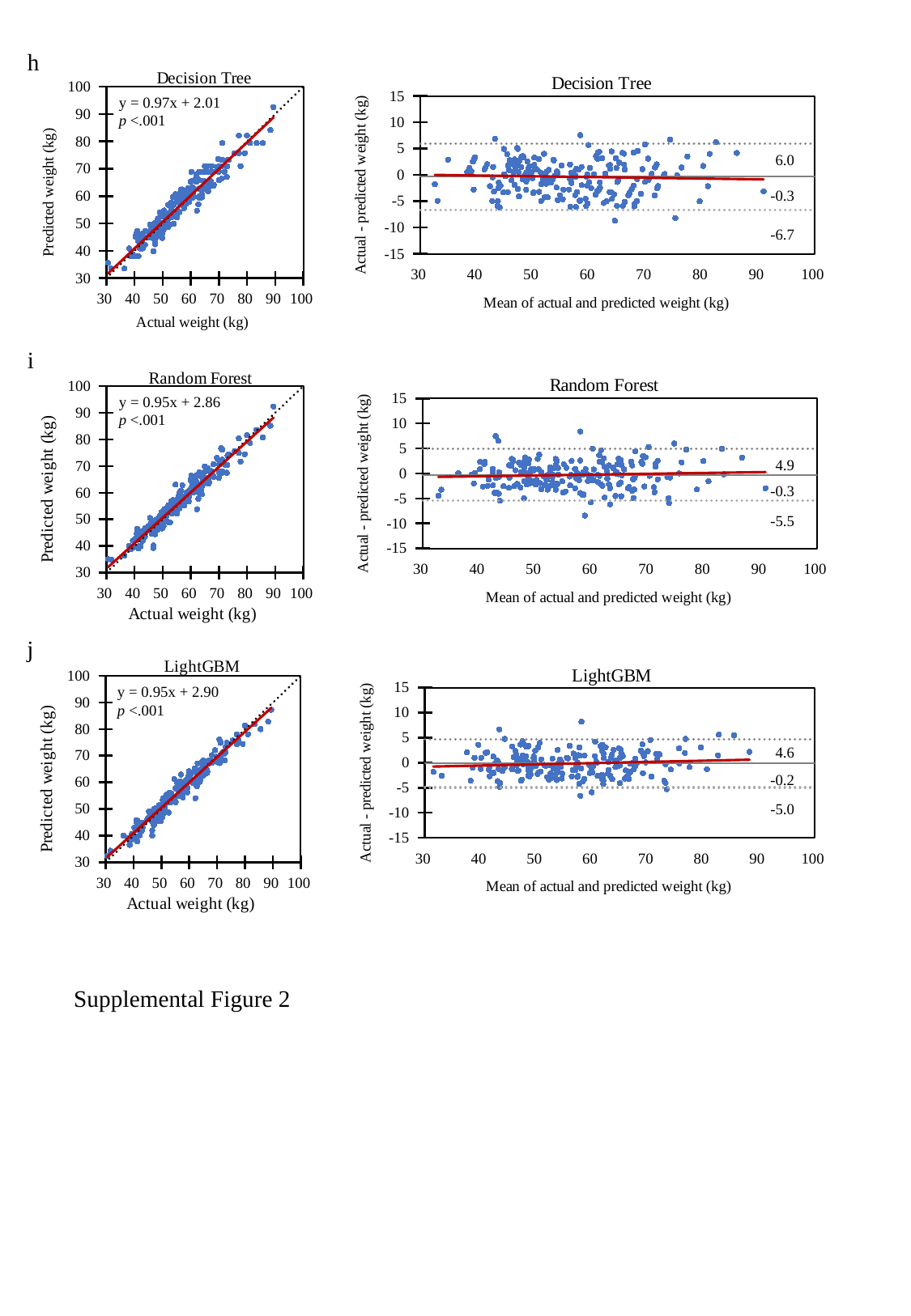

### Chart: Decision Tree
| Category | Mean | |
|---|---|---|
### Chart: Decision Tree
| Category | Mean | | | |
|---|---|---|---|---|h
y = 0.97x + 2.01
p <.001
6.0
-0.3
-6.7
### Chart: Random Forest
| Category | Mean | |
|---|---|---|i
### Chart: Random Forest
| Category | Mean | | | |
|---|---|---|---|---|y = 0.95x + 2.86
p <.001
4.9
-0.3
-5.5
j
### Chart: LightGBM
| Category | Mean | |
|---|---|---|
### Chart: LightGBM
| Category | Mean | | | |
|---|---|---|---|---|y = 0.95x + 2.90
p <.001
4.6
-0.2
-5.0
Supplemental Figure 2

## Slide 5
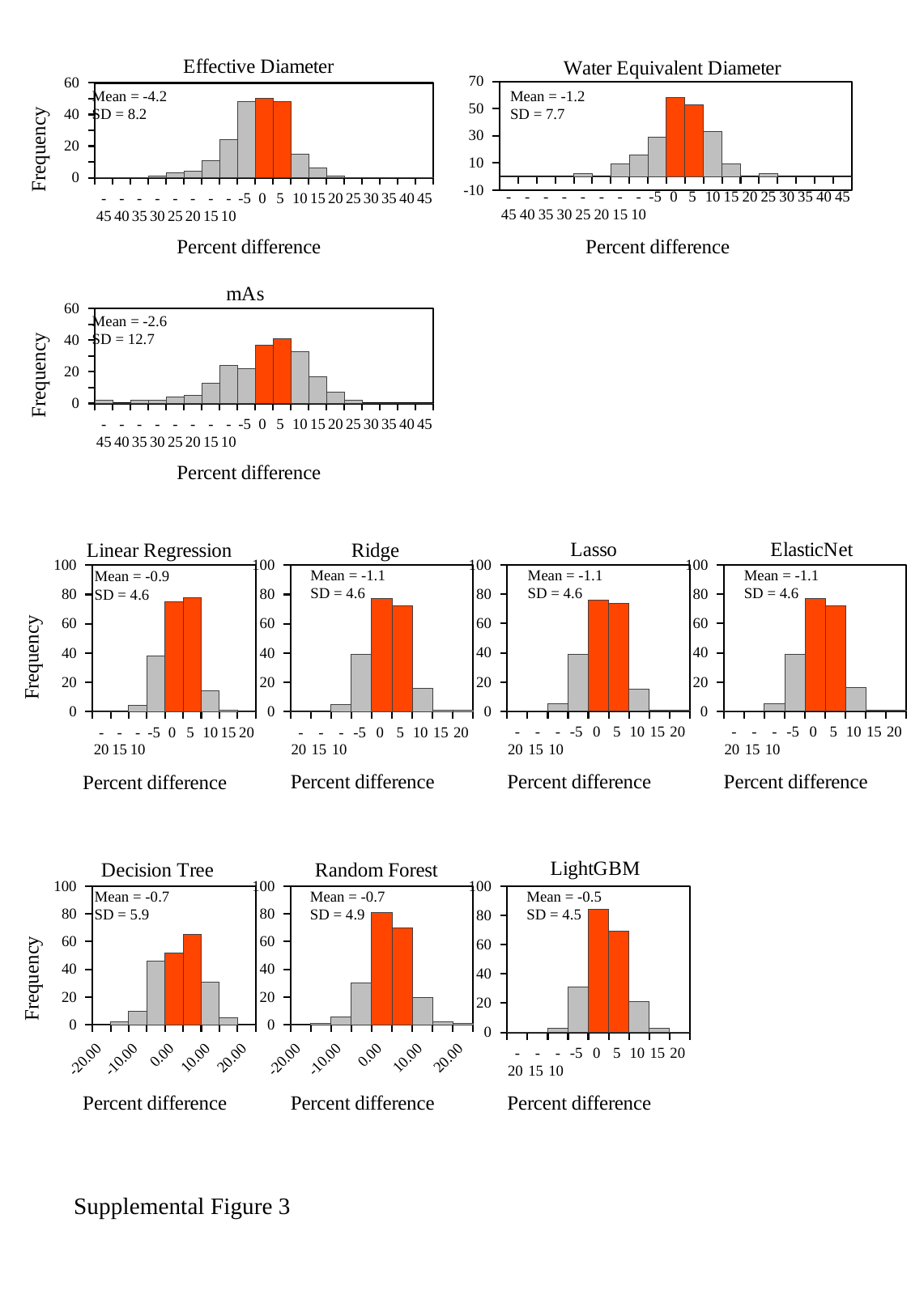

### Chart: Effective Diameter
| Category | |
|---|---|
| -45 | 0.0 |
| -40 | 0.0 |
| -35 | 0.0 |
| -30 | 1.0 |
| -25 | 3.0 |
| -20 | 4.0 |
| -15 | 11.0 |
| -10 | 24.0 |
| -5 | 48.0 |
| 0 | 50.0 |
| 5 | 48.0 |
| 10 | 15.0 |
| 15 | 6.0 |
| 20 | 1.0 |
| 25 | 0.0 |
| 30 | 0.0 |
| 35 | 0.0 |
| 40 | 0.0 |
| 45 | 0.0 |
### Chart: Water Equivalent Diameter
| Category | |
|---|---|
| -45 | 0.0 |
| -40 | 0.0 |
| -35 | 0.0 |
| -30 | 0.0 |
| -25 | 2.0 |
| -20 | 0.0 |
| -15 | 9.0 |
| -10 | 16.0 |
| -5 | 29.0 |
| 0 | 58.0 |
| 5 | 53.0 |
| 10 | 33.0 |
| 15 | 9.0 |
| 20 | 0.0 |
| 25 | 2.0 |
| 30 | 0.0 |
| 35 | 0.0 |
| 40 | 0.0 |
| 45 | 0.0 |Mean = -4.2
SD = 8.2
Mean = -1.2
SD = 7.7
### Chart: mAs
| Category | |
|---|---|
| -45 | 2.0 |
| -40 | 0.0 |
| -35 | 2.0 |
| -30 | 2.0 |
| -25 | 4.0 |
| -20 | 5.0 |
| -15 | 13.0 |
| -10 | 24.0 |
| -5 | 22.0 |
| 0 | 37.0 |
| 5 | 41.0 |
| 10 | 33.0 |
| 15 | 17.0 |
| 20 | 7.0 |
| 25 | 2.0 |
| 30 | 0.0 |
| 35 | 0.0 |
| 40 | 0.0 |
| 45 | 0.0 |Mean = -2.6
SD = 12.7
### Chart: Lasso
| Category | |
|---|---|
| -20 | 0.0 |
| -15 | 0.0 |
| -10 | 5.0 |
| -5 | 39.0 |
| 0 | 76.0 |
| 5 | 74.0 |
| 10 | 15.0 |
| 15 | 1.0 |
| 20 | 1.0 |
### Chart: ElasticNet
| Category | |
|---|---|
| -20 | 0.0 |
| -15 | 0.0 |
| -10 | 5.0 |
| -5 | 39.0 |
| 0 | 77.0 |
| 5 | 72.0 |
| 10 | 16.0 |
| 15 | 1.0 |
| 20 | 1.0 |
### Chart: Ridge
| Category | |
|---|---|
| -20 | 0.0 |
| -15 | 0.0 |
| -10 | 5.0 |
| -5 | 39.0 |
| 0 | 77.0 |
| 5 | 72.0 |
| 10 | 16.0 |
| 15 | 1.0 |
| 20 | 1.0 |
### Chart: Linear Regression
| Category | |
|---|---|
| -20 | 0.0 |
| -15 | 0.0 |
| -10 | 4.0 |
| -5 | 38.0 |
| 0 | 75.0 |
| 5 | 78.0 |
| 10 | 14.0 |
| 15 | 1.0 |
| 20 | 0.0 |Mean = -1.1
SD = 4.6
Mean = -1.1
SD = 4.6
Mean = -1.1
SD = 4.6
Mean = -0.9
SD = 4.6
### Chart: Decision Tree
| Category | |
|---|---|
| -20 | 0.0 |
| -15 | 2.0 |
| -10 | 10.0 |
| -5 | 46.0 |
| 0 | 52.0 |
| 5 | 65.0 |
| 10 | 31.0 |
| 15 | 5.0 |
| 20 | 0.0 |
### Chart: Random Forest
| Category | |
|---|---|
| -20 | 0.0 |
| -15 | 1.0 |
| -10 | 6.0 |
| -5 | 30.0 |
| 0 | 81.0 |
| 5 | 70.0 |
| 10 | 20.0 |
| 15 | 2.0 |
| 20 | 1.0 |
### Chart: LightGBM
| Category | |
|---|---|
| -20 | 0.0 |
| -15 | 0.0 |
| -10 | 3.0 |
| -5 | 31.0 |
| 0 | 84.0 |
| 5 | 69.0 |
| 10 | 21.0 |
| 15 | 3.0 |
| 20 | 0.0 |Mean = -0.7
SD = 5.9
Mean = -0.7
SD = 4.9
Mean = -0.5
SD = 4.5
Supplemental Figure 3
