# Supplementary material for: Machine learning‐based estimation of patient body weight from radiation dose metrics in computed tomography
Source: J Appl Clin Med Phys. 2024 Jul 23;25(9):e14467. doi: 10.1002/acm2.14467 (PMC11492421; doi:10.1002/acm2.14467)
Supplement: Supplementary file 2 — Supporting information [file ACM2-25-e14467-s001.docx]

**Supplementary Table 1.** Summary of the hyperparameter tuning used in seven body weight estimation machine-learning models

| Model | Hyperparameter tuning description |
| --- | --- |
| Linear regression | No critical hyperparameters required tuning |
| Ridge | alpha = 3; random_state = 0 |
| LASSO | alpha = 0.01; random_state = 0 |
| Elastic net | alpha = 0.001; l1_ratio = 0.3; random_state = 0 |
| Decision trees | criterion = “squared_error”; max_depth = 8; min_samples_split = 18; random_state = 0 |
| Random forest | n_estimators = 280; criterion = “gini”; max_depth = 16; min_samples_split = 2; random_state = 0 |
| LightGBM | boosting_type='gbdt; num_leaves = 7; max_depth = 2; learning_rate = 0.1; n_estimates = 220; random_state = 0 |
